# Peritoneal Tuberculosis Mimicking Peritoneal Carcinomatosis

**DOI:** 10.1155/2014/436568

**Published:** 2014-03-04

**Authors:** Mehmet Akce, Sarah Bonner, Eugene Liu, Rebecca Daniel

**Affiliations:** Department of Internal Medicine, Saint Joseph Mercy Hospital, Ann Arbor, Michigan, MI 48106, USA

## Abstract

A 67-year-old male presented with fatigue, abdominal pain , and 30-pound weight loss over 3 months. Computerized tomography (CT) abdomen displayed ascites with thickening and enhancement of the peritoneum and mottled nodular appearing as soft tissue consistent with omental caking worrisome for peritoneal carcinomatosis. A paracentesis revealed white blood cell count of 2,500 with 98% lymphocytes and serum ascites albumin gradient of 0.9 g/L. No acid-fast bacilli were seen by microscopic exam and culture was negative. Purified protein derivative skin test (PPD) was negative and CXR did not reveal any infiltrates. Esophagogastroduodenoscopy (EGD) and colonoscopy were unrevealing. The patient underwent exploratory laparotomy with round ligament and peritoneal biopsies that revealed numerous necrotizing granulomas. Acid-fast bacteria Ziehl-Neelsen stain (AFB) of the biopsy specimen revealed single acid-fast bacilli. Treatment for M. tuberculosis was initiated and final culture revealed that mycobacterium tuberculosis was sensitive to Isoniazid, Rifampin, Ethambutol, and Pyrazinamide. After 6 months of treatment, the ascites and peritoneal carcinomatosis resolved.

## 1. Introduction

Extrapulmonary tuberculosis (ETB) comprises 18.7% of all tuberculosis cases in the USA. Peritoneal tuberculosis, which is caused by mycobacterium tuberculosis, is an uncommon form of ETB and is seen only in 4.7% of all ETB cases [1]. Although both primary tuberculosis (PTB) and ETB cases have decreased over time in the USA, the slower decrease in ETB cases caused a relative increase in the ETB compared to PTB. Peritoneal involvement is the sixth most common site of ETB in the USA and usually is a result of hematogenous spread from a pulmonary focus or direct spread from adjacent organs.

## 2. Case Presentation

**Figure 1:**
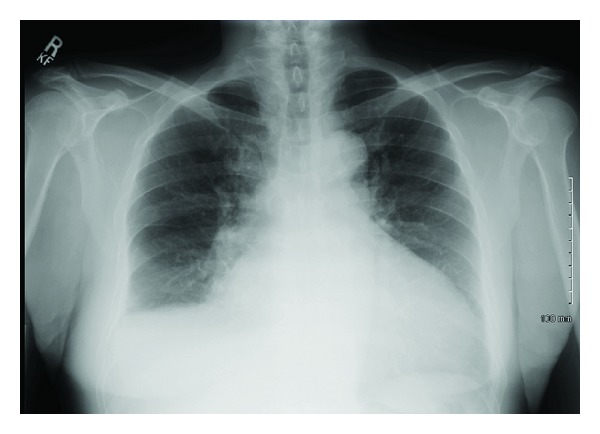
CXR revealed mild blunting of right costophrenic sulcus without consolidation or infiltrates.

**Figure 2:**
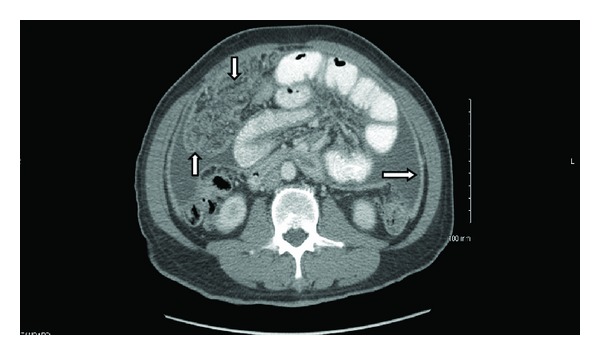
CT abdomen, moderate amount of abdominal, and pelvic ascites with diffuse thickening of peritoneal surfaces. Diffuse thickening also is seen throughout the omentum. Appearance is worrisome for peritoneal carcinomatosis.

**Figure 3:**
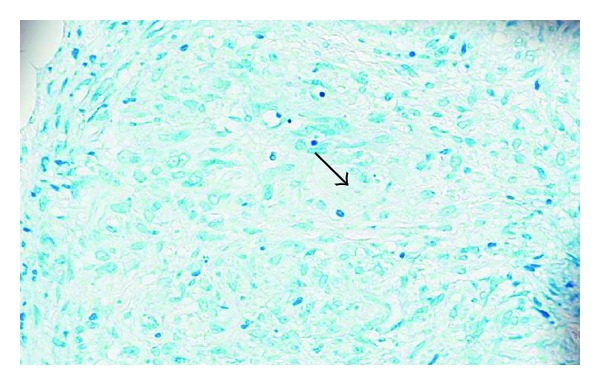
AFB staining of round ligament revealing single acid-fast bacillus consistent with mycobacterium.

**Figure 4:**
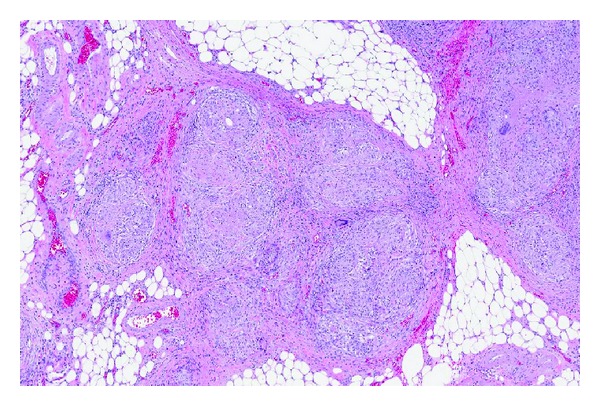
Exploratory laparotomy with round ligament and peritoneal biopsies revealed numerous necrotizing granulomas.

A 67-year-old male presented with fatigue, anemia, and weight loss of 30 pounds in the last 3 months. He denied history of alcohol consumption and endorsed history of travel to Philippines. On physical examination he had pale conjunctiva bilaterally and shifting dullness on abdomen and rest of his physical examination was normal. Initial laboratory studies revealed Hb of 6.1 gm/dL, MCV 58 FL, creatinine 1.50 mg/dL, albumin 3.3 gm/dL, INR 0.77, normal ALT/AST/ALP, and total bilirubin. CXR did not reveal any infiltrates (Figure 1). CT abdomen showed moderate amount of ascites with diffuse thickening of peritoneal surfaces suggestive of peritoneal carcinomatosis (Figure 2). Due to these CT abdomen findings and history of recent weight loss, he underwent a work-up for malignancy with a possible gastrointestinal origin in mind. He underwent diagnostic paracentesis, which revealed WBC of 2,500, with 98% lymphocytes. Cytology was negative for malignancy. No acid-fast bacilli were seen by microscopic exam and culture was negative. PPD was negative. Carcinoembriogenic antigen (CEA) and alpha fetoprotein (AFP) were normal, 1.1 ng/mL and 2.4 IU/mL, respectively. Hepatitis B virus and hepatitis C virus antibodies were negative. EGD revealed duodenal bulbar ulceration, which was ablated, and biopsies were negative for malignancy. Colonoscopy was incomplete due to fixation of the sigmoid colon. Double contrast barium enema was normal. The patient underwent exploratory laparotomy with round ligament and peritoneal biopsies, which revealed numerous necrotizing granulomas. (Figure 3). AFB stain of round ligament revealed single acid-fast bacilli consistent with mycobacterium (Figure 4). PCR analysis was performed and found to be positive for M. tuberculosis complex DNA. Final culture results revealed that mycobacterium tuberculosis was sensitive to Rifampin, Isoniazid, Ethambutol, and Pyrazinamide. He was started on treatment for M. tuberculosis and completed the course. His repeat ultrasound of the abdomen after 6 months showed resolution of ascites and peritoneal carcinomatosis. Currently he is asymptomatic and hemoglobin level has normalized.

## 3. Discussion

The incidence of TB peritonitis among all forms of TB ranges from 0.1% to 0.7% worldwide [2]. The peritoneum is usually involved as a result of hematogenous spread from a pulmonary focus or as a result of direct spread from adjacent organs like the intestine and fallopian tubes. Infection may also result from ingesting contaminated milk or swallowing sputum in the case of active lung disease. Cirrhosis, chronic ambulatory peritoneal dialysis, DM, and HIV are risk factors for peritoneal tuberculosis. The disease is usually subacute with abdominal pain, ascites, and fever being the most common clinical findings [3]. Weight loss, anorexia, malaise, diarrhea, and constipation may also be seen. There are three different clinical forms of peritoneal tuberculosis: wet-ascitic, fibrotic-fixed, and dry-plastic form. The clinical distinction between the three types is not always obvious with the exception of the clinical presentation of abdominal distension, which is not evident in the dry-plastic form. Diagnosis of peritoneal tuberculosis requires a high clinical suspicion since the disease is usually insidious and clinical signs are nonspecific. Imaging showing peritoneal thickening, ascites, omental thickening, and loculations of ascitic fluid is usually helpful for diagnosis. Ascitic fluid analysis, Ziehl-Neelsen stain, polymerase chain reaction assays (PCR), sdenosine deaminase (ADA), T-cell based testing for mycobacterium tuberculosis (ELISPOT), and interferon gamma test are available tests for peritoneal tuberculosis.

The ascitic fluid analysis usually shows lymphocytic predominance and low serum-ascites albumin gradient 1.1 g/L (SAAG); however, this is not a reliable marker of TB and is only helpful to raise the suspicion of TB. Ziehl-Neelsen stain is positive only in about 3% of cases and has very low yield [4]. ADA level above 30 U/L is known to be 94% sensitive in diagnosis of peritoneal tuberculosis; however, the sensitivity is much lower in patients with cirrhosis [2]. By using cut-off values between 36 and 40 IU/L it was shown that the ADA levels had 100% sensitivity and 97% specificity in a meta-analysis of 12 prospective studies [5]. The optimal cut-off point was determined to be 39 IU/L in the same meta-analysis. A study involving 72 patients with suspected extrapulmonary tuberculosis revealed that ELISOT testing had 94% sensitivity and 88% specificity [6]. PCR analysis in body tissues has 95% sensitivity in smear positive patients, but sensitivity is 48% in smear negative patients [2]. In a retrospective analysis of 11 patients presenting with abdominal tuberculosis PCR of ascites fluid was positive in all cases [7]. As only 3% of patients are smear positive PCR sensitivity would be expected to be very low. It is very interesting that our patient had positive PCR of the omental tissue for mycobacterium tuberculosis despite having negative smear of the paracentesis fluid. High interferon gamma concentrations in ascitic fluid have been shown to be valuable in diagnosis of peritoneal tuberculosis [8]. Although ADA level, PCR and interferon gamma levels, and ELISPOT assays are valuable tests in diagnosis of peritoneal tuberculosis, further studies are needed to determine their role in diagnosis of PT.

Only 15–20% of cases have concomitant pulmonary involvement on CXR [4]. The gold standard for diagnosis of peritoneal tuberculosis is growth of mycobacterium tuberculosis from ascitic fluid or peritoneal biopsy specimen [2]. Since the yield of culture from peritoneal fluid is usually low and growth of M. tuberculosis is slow, the diagnosis usually requires laparoscopic or laparotomic peritoneal biopsy [3]. The recommended treatment for peritoneal tuberculosis is conventional antituberculosis therapy for 6 months [9].

## 4. Conclusion

In any patient with several weeks of abdominal pain, weight loss, fever, and lymphocytic dominant ascites with SAAG < 1.1 g/L, TB peritonitis should be considered in differential diagnosis. The yield of smear and culture of peritoneal fluid are low; therefore surgical peritoneal biopsy is helpful and often necessary to prevent the delay in diagnosis and treatment of peritoneal tuberculosis.
